# A Multilayer Interactome Network Constructed in a Forest Poplar Population Mediates the Pleiotropic Control of Complex Traits

**DOI:** 10.3389/fgene.2021.769688

**Published:** 2021-11-12

**Authors:** Huiying Gong, Sheng Zhu, Xuli Zhu, Qing Fang, Xiao-Yu Zhang, Rongling Wu

**Affiliations:** ^1^ College of Science, Beijing Forestry University, Beijing, China; ^2^ College of Biology and the Environment, Nanjing Forestry University, Nanjing, China; ^3^ Center for Computational Biology, College of Biological Sciences and Technology, Beijing Forestry University, Beijing, China; ^4^ Faculty of Science, Yamagata University, Yamagata, Japan; ^5^ Center for Statistical Genetics, The Pennsylvania State University, Hershey, PA, United States

**Keywords:** epistasis, multilayer network, omnigenic model, pleiotropic control, system mapping, variable selection

## Abstract

The effects of genes on physiological and biochemical processes are interrelated and interdependent; it is common for genes to express pleiotropic control of complex traits. However, the study of gene expression and participating pathways *in vivo* at the whole-genome level is challenging. Here, we develop a coupled regulatory interaction differential equation to assess overall and independent genetic effects on trait growth. Based on evolutionary game theory and developmental modularity theory, we constructed multilayer, omnigenic networks of bidirectional, weighted, and positive or negative epistatic interactions using a forest poplar tree mapping population, which were organized into metagalactic, intergalactic, and local interstellar networks that describe layers of structure between modules, submodules, and individual single nucleotide polymorphisms, respectively. These multilayer interactomes enable the exploration of complex interactions between genes, and the analysis of not only differential expression of quantitative trait loci but also previously uncharacterized determinant SNPs, which are negatively regulated by other SNPs, based on the deconstruction of genetic effects to their component parts. Our research framework provides a tool to comprehend the pleiotropic control of complex traits and explores the inherent directional connections between genes in the structure of omnigenic networks.

## Introduction

The study of gene pleiotropy has become a focus of genetic research in recent years. Pleiotropy describes the phenomenon that single genes can have multiple biological effects, so that an individual exhibits multiple traits (Solovieff et al., 2013). Pleiotropy is an important factor in genotype–phenotype transmission (Dudley et al., 2005; Gregory et al., 2012; Geiler-Samerotte et al., 2020), which can help us to understand how the underlying biochemical pathways determine the behavior of the cells in which they are present (Roth et al., 2021). With the development of genome-wide association statistical models, the regulatory roles of genes and their interactive effects have received sustained attention in research on pleiotropy (Sivakumaran et al., 2011; Visscher and Yang, 2016; Watanabe et al., 2019); these existing genetic studies mainly focus on the action of identified key genes, which account for only a small amount of phenotypic variation. The current understanding of the networks of genes that actually drive the development of complex traits, and how genes throughout the genome interact remains inadequate.

In the early 20th century, reductionism had a positive impact on the development of biological understanding (Osler, 1969). Conventional reductionism is the theory that complex systems and phenomena can be understood and described by breaking them down to their fundamental parts. According to the complex network mathematical model, the salient information can be extracted from a network constructed using reductionist principles. However, unlike physico-chemical networks, in biological networks, organisms have an organic character that emerges not through the sum of all components, but the interconnection between them (Regenmortel, 2004; Roukos 2011; Mazzocchi 2012). An “omnigenic” model was therefore proposed to take into account the activity of genes in cells, which form a broad network in which each gene exerts an influence on the occurrence of disease or development of traits, including those without any obvious connection to traits or diseases in interconnected gene networks (Boyle et al., 2017; Wray et al., 2018; Liu et al., 2019).

Omnigenic network modeling has become a powerful and fundamental tool for analyzing interactomes and quantifying relationships among genes; many statistical networking methods have been established (Carter et al., 2004). However, most related gene regulatory networks have their own underlying mathematical rationale and assumptions, thus results lack robustness (Marbach et al., 2012). In addition, an omnigenic network involving large amounts of genomic data is high-dimensional, which brings inevitable challenges in computing. Clustering techniques are required to sort complicated, high-dimensional genes into communities or modules through modularity theory (Wang and Huang, 2014; Huynh-Thu and Sanguinetti, 2019). Based on the dynamic nature of gene behaviors, functional clustering has made it possible to identify the similarity of temporal genetic effects from large numbers of loci, thus resolving biological and computational complexity (Kim et al., 2008; Li et al., 2010; Wang et al., 2012).

In this article, we propose a new model to explore the multilayer interactome network mediating the pleiotropic control of complex traits (in this case tree height and diameter) by integrating system mapping (Wu et al., 2011; Bo et al., 2014; Sun and Wu, 2015), functional clustering, and differential genetic regulatory systems (Jong, 2002; Jong et al., 2003). Given that the metabolism of an organism is a network of interacting processes (Michael, 2019), we established a coupled regulatory interaction (CRI) differential equation model to describe interactions between complex traits growth (Wu et al., 2011; Jérôme et al., 2013). This differential equation can be embedded into the system mapping model to discern specific quantitative trait loci (QTLs) that control the traits, according to differences between genotypes in the equation parameters. Further, we reveal gene interactions, and describe how genes are implicated in the control of intracellular and intercellular processes through multilayer gene network modeling (Someren et al., 2002; Margolin and Califano, 2010; Yukilevich et al., 2010; Costanzo et al., 2019; Wu and Jiang, 2021); this incorporates modularity theory to resolve ultrahigh-dimensional computational complexity. From this, networks of separate modules can be constructed, and modules can be divided into submodules and sub-submodules; genome-wide epistasis can thus be interpreted from an evolutionary game theory perspective (Smith and Price, 1973) postulating that interactions between genes can lead to genetic effect payoff. Our multilayer interactome network provides a powerful computational tool in the mechanistic analysis of large, genome-wide expression datasets and revolutionizes our understanding of the pleiotropic control of complex traits.

## Materials and Methods

### Plant Materials

We used published data from trees as mapping population for our study (Xu et al., 2016). It comprises a full-sib family derived from hybridization between the female clone I-69 of *Populus deltoides* and the male clone I-45 of *Populus* × *euramericana*, which were introduced from the United States in the 1970s (Wu et al., 1992). This hybridization generated 450 hybrid trees, planted with ramets in a uniform land at Zhangji Forest Farm, Xuzhou, Jiangsu, China. The two parents, I-69 and I-45, and 64 randomly-selected hybrids were used for stem growth analysis, in which annual data comprising stem height and stem diameter during the first 24 years of growth from 1987 to 2010 were measured. The trees were genotyped at single nucleotide polymorphism (SNP) sites using the Applied Biosystems QuantStudio 12K Flex Real-Time Polymerase Chain Reaction (PCR) System for genome-wide mapping. 156 362 SNPs were characterized through stringent quality-control filters segregating with different patterns, of which 94 591 SNPs belong to testcross markers and 61 771 SNPs belong to intercross markers, respectively. The testcross markers are those at which one parent is heterozygous whereas anotherr is homozygous. The intercross markers are derived from two heterozygous parents.

### CRI Differential Equations of Complex Traits

The pattern of interactions between tree height and diameter is fundamental for the development and application of many growth and yield models. It is the focus of theoretical and empirical analyses indicating pleiotropic control (Dharmawardhana et al., 2010; Jiang et al., 2016). The growth relationship between diameter and height can be described by many traditional models, such as nonlinear functions and generalized height-diameter functions (Temesgen and Gadow, 2004; Ahmadi and Alavi, 2016; Roya and Tooba, 2020), and mixed-effect models (Sharma and Parton, 2007; Crecente-Campo et al., 2010; Bronisz and Mehttalo, 2020; Liao et al., 2020), which evaluate the overall change and trends among traits by establishing the relation of function. However, the internal coordination of tree height and diameter can be understood in more depth by investigating the underlying biological mechanisms of their control. From the perspective of game theory, we introduced the Lotka–Volterra differential equation (May, 1975) to represent the specific forms of the interaction between the complex traits of growth in stem diameter and height. We present a coupled regulatory interaction (CRI) differential equation to describe the growth relationship between traits, which separates the growth of these traits into independent and dependent parts:

$$\{\begin{array}{c} \frac{dH}{dt}=\alpha_{H}\left(1-\frac{H}{K_{H}}\right)H+\alpha_{H}\beta_{H\leftarrow D}HD=\hat{H}+I_{H\leftarrow D}\\ \frac{dD}{dt}=\alpha_{D}\left(1-\frac{D}{K_{D}}\right)D+\alpha_{D}\beta_{D\leftarrow H}DH=\hat{D}+I_{D\leftarrow H} \end{array}\;\;\;\;\;\;\;\;\;\;\;$$ (1)

where $\hat{H}$ or $\hat{D}$ is the independent growth of each trait, determined by its intrinsic properties, and $I_{H\leftarrow D}$ or $I_{D\leftarrow H}$ describes the interactive growth of each trait, depending on how it interacts with the other, coexisting trait. H and D in this study represent the growth of stem height and diameter, $\alpha_{H}$ and $\alpha_{D}$ represent the growth rate, and $K_{H}$ and $K_{D}$ represent the asymptotic values. $\beta_{H\leftarrow D}$ and $\beta_{D\leftarrow H}$ are dependent parameters; the size of the positive or negative dependent parameters $\beta_{H\leftarrow D}$ and $\beta_{D\leftarrow H}$ indicate the type of interactive relationship. Specifically, when the dependent parameter is positive, the growth is promoted by the coexistence characteristics. Conversely, when the dependent parameter is negative, the growth is hindered. If the dependent parameter is zero, the overall growth depends solely on independent growth, which means that there is no interaction between the two traits. The interaction of the two traits can be described by a strategy set, shown in Table 1 and summarized as follows:

• neutral interaction strategy: no interaction between the two characteristics;
• cooperative interaction strategy: the growth of one characteristic is promoted by the other, without hindering the growth of the latter;
• antagonistic interaction strategy: the overall growth of at least one characteristic is inhibited by the other.

**TABLE 1:** A strategy set of stem growth on traits interaction. The regulation strategy table formed by the different positive and negative combinations of two interactive regulation parameters. 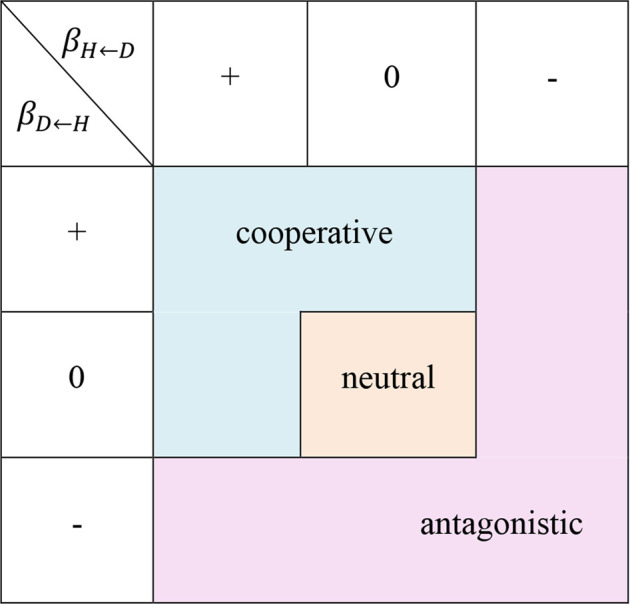

The parameters in the CRI differential equation describe the developmental mechanisms behind the formation and expression of the two traits and their interaction. Using this CRI differential equation, we can explore the dynamic changes of the growth of each trait, and quantitatively analyze the nature of the interaction between traits.

### Identification of Interacting QTLs and Trait Regulation

Systems mapping is a classical approach for mapping complex traits by comparing the genotypic differences in growth equation parameters throughout the genome (Wu et al., 2011; Bo et al., 2014; Sun and Wu, 2015). We designed a mapping population of *n* trees. Genome-wide SNPs were genotyped in all trees using high-throughput methods, and trees were phenotyped for height and diameter at a series of time points (1,⋯,T) during development. The phenotypic values of tree i (i=1,⋯,n) for height and diameter are expressed as:

$y_{1i}=\left(y_{1i}\left(1\right),\cdots ,y_{1i}\left(T\right)\right)$ and $y_{2i}=\left(y_{2i}\left(1\right),\cdots ,y_{2i}\left(T\right)\right)$ .

The joint likelihood for n observations can be expressed as:

$$L\left(y_{1},y_{2}\right)=\prod_{i=1}^{n}f\left(y_{1i},y_{2i};\text{Θ},\psi \right)\;$$ (2)

where $f\left(y_{1i},y_{2i};\text{Θ},\psi \right)$ is a probability density function of bivariate normal distribution with mean vector described as:

$$\mu =\left(\mu_{1}\left(1\right),\cdots ,\mu_{1}\left(T\right);\mu_{2}\left(1\right),\cdots ,\mu_{2}\left(T\right)\right)$$

represented by the parameters:

$$\text{Θ}=\left(\alpha_{H},K_{H},\beta_{H\leftarrow D},\alpha_{D},K_{D},\beta_{D\leftarrow H}\right)$$

and the covariance matrix:

$$\text{Σ}=\left(\begin{array}{cc} {\text{Σ}}_{1} & {\text{Σ}}_{12}\\ {\text{Σ}}_{21} & {\text{Σ}}_{2} \end{array}\right)$$

where the diagonal elements are the variance matrices of each trait, and the off-diagonal elements are the covariance matrices between a pair of traits. We used a first-order structured antedependent (SAD (1)) statistical model controlled by a set of specific parameters ψ to express the longitudinal covariance matrix (Zhao et al., 2005a; Zhao et al., 2005b).

Considering the difference in genotype on the growth of trees, we constructed the likelihood function:

$$L_{1}\left(y_{1},y_{2}\right)=\prod_{j=1}^{J}\prod_{i=1}^{n_{j}}f_{j}\left(y_{1i},y_{2i};{\text{Θ}}_{j},\psi \right)\;$$ (3)

where *J* is the number of QTL genotypes and $n_{j}$ is the number of those trees carrying genotype j, satisfying:

$$\sum_{j=1}^{J}n_{j}=n.$$

$f_{j}\left(y_{1i},y_{2i};{\text{Θ}}_{j},\psi \right)$ is a probability density function of bivariate normal distribution with mean vector defined as:

$$\mu_{j}=\left(\mu_{j1}\left(1\right),\cdots ,\mu_{j1}\left(T\right);\mu_{j2}\left(1\right),\cdots ,\mu_{j2}\left(T\right)\right)$$

represented by parameters:

$${\text{Θ}}_{j}=\left(\alpha_{Hj},K_{Hj},\beta_{H\leftarrow Dj},\alpha_{Dj},K_{Dj},\beta_{D\leftarrow Hj}\right)$$

and covariance matrix Σ.

We incorporated the simplex (Zhao et al., 2004), expectation maximization (EM) (Dempster, 1977), and fourth-order Runge–Kutta algorithms to obtain maximum likelihood estimates (MLEs) of the parameters in the mean vector and covariance matrix, respectively. Based on the likelihood of Eqs 2, 3, we can test whether a given SNP is significantly associated with trait allometry, using the following formula:

$H_{0}:\text{Θ}\equiv {\text{Θ}}_{j}$ versus $H_{1}:{\text{Θ}}_{j}\neq \text{Θ}$ , for j=1,⋯,J

In which the log likelihood ratio is calculated and compared with a genome-wide critical threshold. When the null hypothesis above is rejected, this means that significantly associated QTLs have been detected. These QTLs can be further tested to determine whether they affect the independence and interdependence of traits:

$$H_{0}:\;\left(\alpha_{Hj},K_{Hj},\alpha_{Dj},K_{Dj}\right)=\left(\alpha_{H},K_{H},\alpha_{D},K_{D}\right),for\;j=1,\cdots ,J$$

$$H_{0}:\;\left(\beta_{H\leftarrow Dj},\beta_{D\leftarrow Hj}\right)=\left(\beta_{H\leftarrow D},\beta_{D\leftarrow H}\right),for\;j=1,\cdots ,J$$

### Modules Detection From Bivariate Functional Clustering

For all *p* SNPs throughout the genome, we calculated the genetic standard deviation based on the parameters from maximum likelihood estimation for both traits, to describe the genetic effect of SNPs on trait development. We utilized functional clustering to identify distinct patterns of gene expression dynamics by dividing *p* SNPs into L tight-knit modules (Kim et al., 2008; Li et al., 2010; Wang et al., 2012). In our research, functional clustering is extended into bivariate functional clustering including height and diameter. The following equations denote the vectors of genetic effects of SNP k (k=1,⋯,p) on height and diameter, respectively:

$$g_{1k}=\left(g_{1k}\left(1\right),\cdots ,g_{1k}\left(T\right)\right)$$

$$g_{2k}=\left(g_{2k}\left(1\right),\cdots ,g_{2k}\left(T\right)\right)$$

The likelihood based on a mixture model is formulated as:

$$L_{2}\left(g_{1},g_{2}\right)=\prod_{k=1}^{p}\sum_{k=1}^{L}\left[\omega_{l}f_{l}\left(g_{1k},g_{2k};{\text{Φ}}_{l}\right)\right]\;$$ (4)

where $\omega_{l}$ is a prior probability representing the proportion of module *l,* and satisfying $\sum_{l=1}^{L}\omega_{l}=1$ , $f_{l}\left(g_{1k},g_{2k};{\text{Φ}}_{l}\right)$ is a probability density function of bivariate normal distribution with ${\text{Φ}}_{l}$ as a vector of unknown parameters structuring the cluster-specific mean vector:

$$u_{l}=\left(u_{l1}\left(1\right),\cdots ,u_{l1}\left(T\right);u_{l2}\left(1\right),\cdots ,u_{l2}\left(T\right)\right)$$

and covariance matrix ${\text{Σ}}_{g}$ .

We incorporated nonparametric Legendre orthogonal polynomials (LOP) mathematical equation and the SAD (1) statistical model to fit the mean-covariance structures. A hybrid EM-simplex algorithm was implemented to estimate the parameters ${\text{Φ}}_{l}$ in likelihood of Eq. 4; the posterior probability that SNP k belonging to a particular module *l* in each iteration can be determined as:

$${\text{Ω}}_{l|k}=\frac{\omega_{l}f_{l}\left(g_{1k},g_{2k};{\text{Φ}}_{l}\right)}{\sum_{l^{'}}^{L}\omega_{l^{'}}f_{l^{'}}\left(g_{1k},g_{2k};{\text{Φ}}_{l^{'}}\right)}$$

and the proportion of module *l* is calculated by:

$$\omega_{l}=\frac{\sum_{k=1}^{p}{\text{Ω}}_{l|k}}{p}$$

An optimal number of SNP clusters in terms of their different genetic effects can be determined using penalized likelihood criteria, such as AIC and BIC.

### Network Construction

Molecular-level genetic regulatory systems can help us to understand how genes are implicated in trait growth processes through networks. A universal property of complex networks is that the change of one component (usually expressed in the form of a rate equation) in the system is a function of other components:

$$\frac{dx_{l}}{dt}=G_{l}\left(x\right),\;1\leq l\leq L$$

where $x={\left[x_{1},\cdots ,x_{L}\right]}^{\prime }$ is the vector of system components, and $G_{l}:\;{\mathbb{R}}^{n}\to \mathbb{R}$ is the function (generally non-linear) that determines the dynamics model of the entire system (Jong, 2002).

Network theory states that the observed value of a variable is the sum of the components of its own strategy and those derived from the strategies of its interactive counterparts (Wu and Jiang, 2021). The relational structure of each component can be divided into independence and dependence, to explore how these SNPs interconnect and interdepend. Where $g_{1l}=\left(g_{1l}\left(1\right),\cdots ,g_{1l}\left(T\right)\right)$ and $g_{2l}=\left(g_{2l}\left(1\right),\cdots ,g_{2l}\left(T\right)\right)$ denote the vectors of overall genetic effects of module *l* on height and diameter, respectively, we can derive an ordinary differential equation (ODE)-based equation system, as follows:

$$\frac{dg_{\cdot l}}{dt}=G_{l}^{0}\left(g_{\cdot l};{\text{Θ}}_{l}\right)+\sum_{l^{\prime }=1,l^{\prime }\neq l}^{L}G_{l\leftarrow l^{\prime }}\left(g_{\Delta l^{\prime }};{\text{Θ}}_{l\leftarrow l^{\prime }}\right),l=1,\cdots ,L$$ (5)

where $g_{\cdot l}$ is the net genetic effect of module *l* on height or diameter. This can be deconstructed into two components: $G_{l}^{0}\left(g_{\cdot l};{\text{Θ}}_{l}\right)$ is a time-varying function that characterizes the independent genetic effect of module *l* with the assumption that it occurs in isolation; and $\sum_{l^{\prime }=1,l^{\prime }\neq l}^{L}G_{l\leftarrow l^{\prime }}\left(g_{\cdot l^{\prime }};{\text{Θ}}_{l\leftarrow l^{\prime }}\right)$ is a time-varying function that characterizes the dependent genetic effect of module *l* that arises from the influence of the other module $l^{\prime }$ . ${\text{Θ}}_{l}$ and ${\text{Θ}}_{l\leftarrow l^{\prime }}$ are the sets of parameters that fit the independent and dependent functions, respectively.

The established procedure for constructing genetic network consists of the following three steps: data smoothing, variable selection, and ODE solving. We used LOP to smooth the independent and dependent functions; by interpolating additional values on the curve of best fit of genetic effects over time, the case that the number of modules may be larger than the number of time points can be solved. The network sparsity theory states that there is a limit to the number of links to maintain the stability of the network structure (Liu et al., 2011; Allesina and Tang, 2012; Michailidis and Alché-Buc, 2013; Busiello et al., 2017). It is necessary to filter modules through variable selection from the regression model:

$$g_{\cdot l}\left(t\right)=a_{l}+\sum_{l^{\prime }=1,l^{\prime }\neq 1}^{L}b_{l^{\prime }}g_{\cdot l^{\prime }}\left(t\right)+e_{l}\left(t\right)$$

where $a_{l}$ is the constant, $b_{l^{\prime }}$ is the regression coefficient of variable $\;l^{\prime }$ , and $\;e_{l}\left(t\right)$ is the residual error. Here, we incorporated LASSO-based variable selection (Tibshirani, 1996) to choose a set of the most significant dependent modules for a focal module. Lastly, we used the fourth-order Runge–Kutta algorithm to solve the simplified ODEs (5), and we calculated the directional, weighted interactions among modules.

## Results

### Growth Trajectories and Temporal Patterns of Genetic Effects Identified Through System Mapping

**FIGURE 1:**
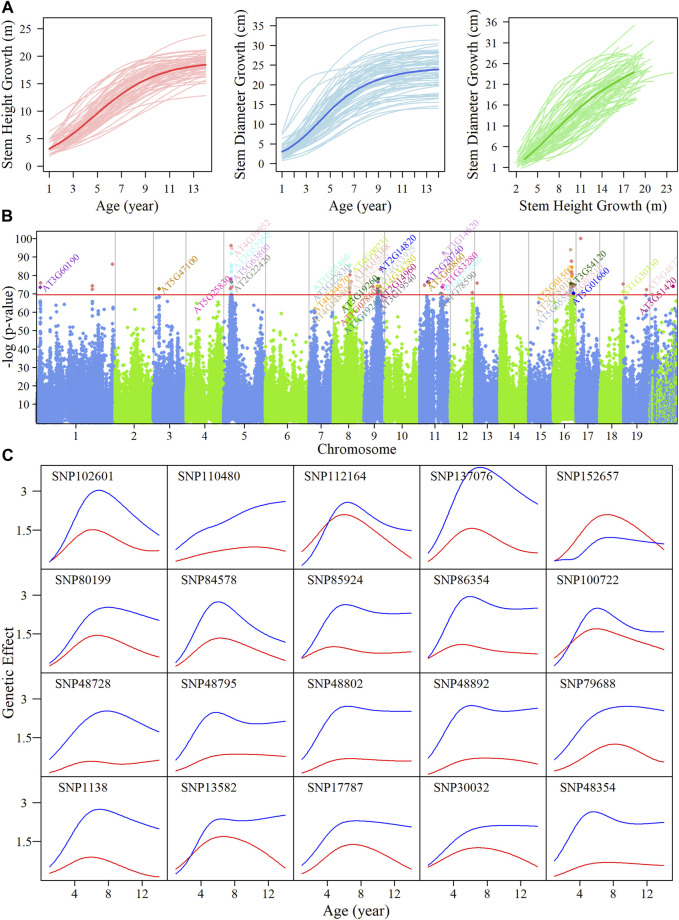
System mapping for the identification of significant single nucleotide polymorphisms (SNPs) goverining growth in stem height and diameter in an interspecific, full-sib family of *Populus* during the first 14 years. **(A)** Overall growth trajectories for stem height (red lines) and diameter (blue lines) fitted using a CRI equation, and relationship curves between traits (green lines) in hybrid poplars. The mean curves for the two growth traits are indicated by darker colored lines. **(B)** Manhattan plot of *p* values after FDR correction over 19 chromosomes of the *Populus* genome by system mapping. Horizontal lines represent the critical threshold at the $10^{-25}$ significance level obtained after Bonferroni correction. The annotations in the significant region are genes with known biological function. **(C)** Genetic effect curves of 20 distinct significant SNPs identified from the Manhattan plot of stem height (red) and diameter (blue).

In this paper, we used forest poplar annual growth data for stem height and diameter from 1 to 14 years. The growth curves of traits over time follow a sigmoid curve. Many classical growth equations provide quantitative assessment to capture biological rule, such as Gompertz (Gompertz, 1815), Korf (Lundqvist, 1957) and Richards (Richards, 1959). However, these classical models can only evaluate the overall change of one trait as a function of the other, but does not provide insight into the internal mechanisms of how height growth affects diameter growth or vice versa. Given the possible interactions between diameter and height, we used the CRI differential equation to fit the trait growth $\left(R^{2}>0.99\right)$ (Figure 1A). Estimated parameters and statistical evaluation values for the Gompertz, Korf, and Richards classical growth equations and the CRI differential equation to fit the average growth curve are shown in Supplementary Table S1. This permitted a comparison of the accuracy and complexity of each equation, which showed that the CRI differential equation had the best fit. The residuals of the growth data were randomly distributed on the predicted value (Supplementary Figure S1), indicating that the CRI differential equation was robust. The growth pattern of stem height and diameter follows an antagonistic interaction strategy (Supplementary Figure S2). Growth in stem height is inhibited by growth in stem diameter, dramatically reducing the overall growth in stem height; conversely, growth in stem height was found to promote stem diameter growth, indicating an interactive effect between the two traits.

We implemented system mapping to quantitatively analyze the interaction between diameter and height (Figure 1B) based on the bivariate normal distribution model; the identified significant quantitative trait loci (QTLs) reveal the physiological mechanism of competitive or cooperative strategies. A total of 88 intercross SNPs and 17 testcross SNPs were found to significantly regulate the interaction in growth of two traits. Over half of these SNPs were within, or adjacent to, candidate genes involving the functions of plant growth-related pathways. For example, SNP 30032 on chromosome 3 was found to be in a region of the calcineurin B-like protein, CBL9 (Pandey et al., 2004), which regulates phytohormone abscisic acid (ABA) responses.

Detailed information on these SNPs that were significantly associated with our traits of interest, including segregation types and physical positions, are given in Supplementary Table S2. A majority of the SNPs were found to be located on chromosomes 5, 8, 9, 11, and 16. SNPs that are highly linked on the same chromosome are likely to represent the same QTL, collectively. We then explored how these QTLs percolate through the entire regulatory network structure. The temporal pattern of genetic effects exerted by the QTLs we identified was calculated, as shown in Supplementary Figure S3; almost all of these SNPs had a stronger effect on diameter compared with height, except for SNP 152657. The temporal pattern of QTLs effects on trait growth varied, but most QTLs had similar genetic effect patterns, in which effects increased initially and then decreased. We analyzed the dynamic genetic correlations on stem height and diameter indicating the QTLs with pleiotropy effects (Supplementary Figure S4). We chose 20 distinct QTLs randomly, as shown in Figure 1C; the effect pattern of SNP 110480 was to keep enhancing with growing time, SNP 112164 and SNP 100722 were found to be responsible for both height and diameter growth with similar intensity, and for SNP 137076, there was a marked difference in effect values between the two traits.

### Network Modules of Genetic Effect Dynamics Based on Omnigenic Theory

The detection of individual QTLs by system mapping has provided the first detailed understanding of the genetic basis of complex traits, but it may provide limited insight into how common SNPs across the genome, which are below the threshold for statistical significance, act and interact to regulate growth traits (Yang et al., 2010). Some common SNPs are not necessarily significantly associated with traits by themselves, but play an important role in regulating other loci and are therefore indirectly involved in trait control. To explore the genetic contribution from these common SNPs that may have been missed, we carried out a quantitative analysis of epistatic effects among genome-wide SNPs in omnigenic networks. Based on our CRI differential model, we estimated the effect on stem height and diameter of each SNP through system mapping.

The use of high-dimensional genome-wide SNP datasets are essential to revealing how all interconnected genes generate or regulate the expression of complex traits and pleiotropic control. The regulatory networks of gene pleiotropy in living organisms can be usefully compared the vast networks of stars in interstellar clusters, which in turn form galaxies, and then superclusters of galaxies. Although it is computationally complex, dimensions can be reduced by cluster. We incorporated modularity theory (Newman, 2006; Cantini et al., 2015) into network modeling in which nodes are densely connected in modules, with sparser connections between modules. In this paper, using a galactic analogy, we constructed metagalactic networks, intergalactic networks, and local interstellar networks to describe the layers of structure between modules, submodules and individual SNPs, respectively.

**FIGURE 2:**
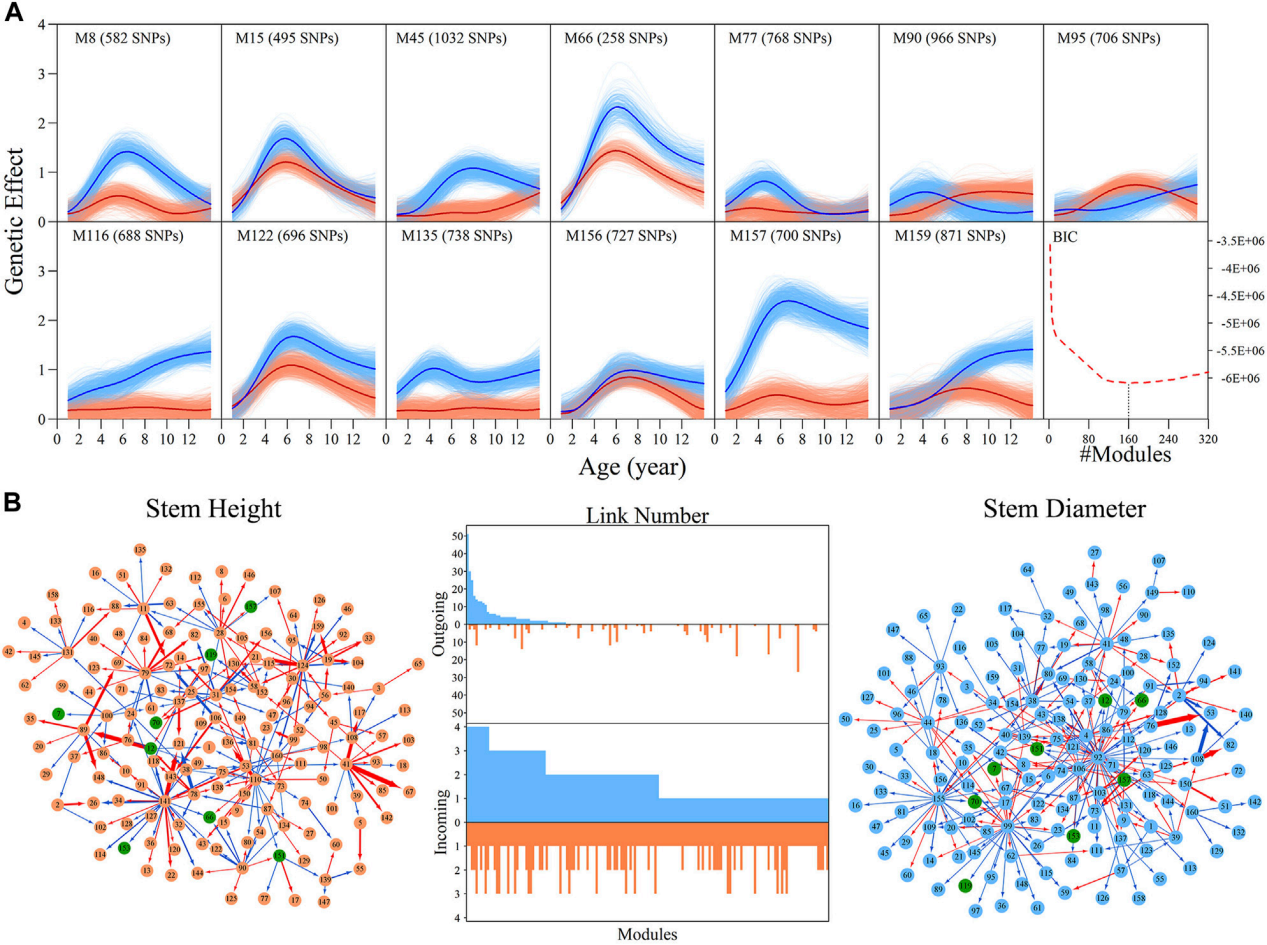
Genetic effect clusters and metagalactic network modules for stem height and stem diameter growth in an interspecific full-sib family of *Populus*. **(A)** Genetic effect curves for 17 representative modules of stem height (red) and diameter (blue) chosen from a total of 160 gene modules detected by bivariate functional clustering; Bayesian Information Criterion analysis showed 160 as the optimal number of modules. **(B)** Metagalactic genetic networks containing 160 modules reconstructed using the mean effect values of each module for height and diameter, where red and blue arrowed lines denote inhibition and activation effects, respectively. The thickness of lines is proportional to the strength of the regulatory interaction. Modules containing quantitative trait loci (QTLs) are highlighted by green points. The distribution of the number of outgoing links and incoming links across 160 modules for height (red, bottom) and diameter (blue, top) is enumerated between networks.

Genome-wide SNPs can be classified into different modules based on similarities in the pattern of genetic effects. We implemented bivariate functional clustering to classify the height and diameter effects of 156,362 SNPs into different modules. According to the comparison of BIC values for different cluster numbers, the most parsimonious number of modules was found to be 160. Each module represents a specific temporal pattern of genetic effects on the growth of height and diameter, which differs from those from other modules (Supplementary Figure S3). In Figure 2A, 13 representative modules are illustrated, which suggest pronounced discrepancies exist in the temporal patterns of genetic effects on the growth of both height and diameter. Some modules, such as M90 and M95, have greater genetic effects on height growth than diameter for a period of time, but some modules display an inverse pattern. All these differences in the time-dependent change of genetic effects contribute to the pleiotropic effects of the genetic architecture on growth in stem height and diameter.

We then explored how these 160 distinct modules are interconnected. We calculated the mean genetic effect curve for each module to construct metagalactic genetic interaction networks among modules (Figure 2B), where nodes represent the collective effect of all SNPs within a module. This showed that both the height and diameter networks are highly sparse; directional positive and negative epistasis together dominate in the pairwise links. In both the height and diameter networks, positive epistasis constitutes a larger portion of the links: 51.04% in the height and 57.93% in the diameter growth networks, suggesting that genes tend to cooperate in the growth of these traits. Several negative links between modules indicated epistatic inhibition with a great strength, such as M41→M67 in the height network and M76→M53 in the diameter network. Outgoing and incoming links describe the activation or inhibition one module exerts on another, and the activation or inhibition of another module on the module of interest, respectively. We counted the total number of outgoing and incoming links for each module in the network, and plotted the distribution (Figure 2B, middle panel). The numbers of outgoing links differed greatly across modules, ranging from 0 to 50; only a small subgroup of highly-interconnected modules predominated in the genetic network, and most modules were relatively minor nodes. The distribution of incoming links was much more consistent between modules. As shown in Figure 2B, we found that the character of the module is intricate, with outgoing and incoming links that varied between height and diameter networks: some nodes that were predominant in the height network, such as M141, were minor nodes in the diameter network. Conversely, modules such as M92 were predominant in the diameter network but minor in the height network. Some modules, such as M41, were also predominant in both networks.

### QTL Deconstruction in Multilayer Network Architecture

**FIGURE 3:**
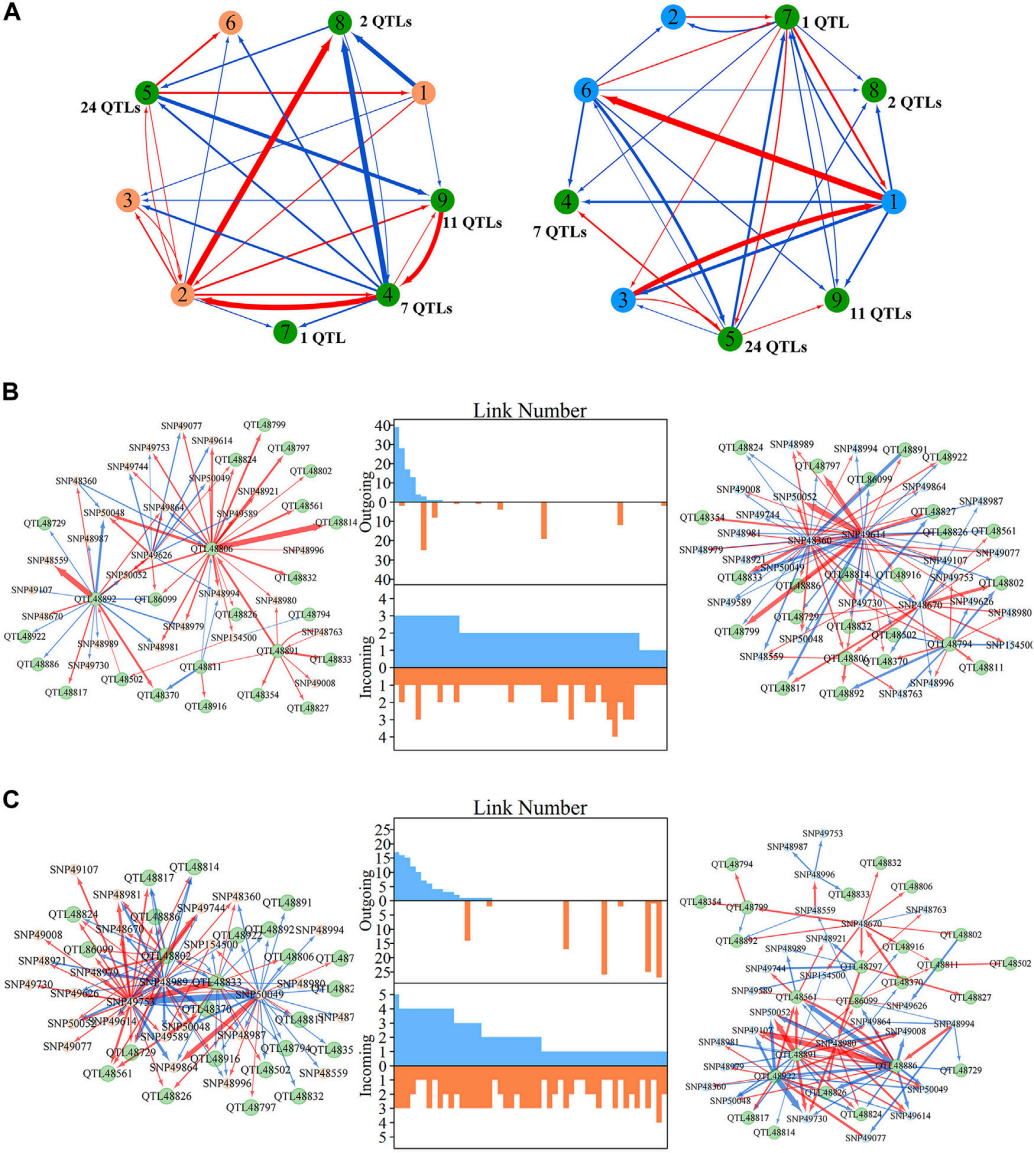
Intergalactic genetic networks of quantitative trait loci (QTL)-containing submodules and local interstellar networks of individual single nucleotide polymorphisms (SNPs) for stem height and diameter. **(A)** Genetic networks among nine submodules of module M153. **(B)** Genetic networks among 50 SNPs from submodule 5, SM5/M153. **(C)** Independent genetic networks among 50 SNPs from submodule 5, SM5/M153. Red and blue arrowed lines represent inhibition and activation, where the thickness of lines is proportional to the strength of regulation. The submodules containing each QTL are highlighted by green points, and QTLs are highlighted within the intergalactic networks. The distribution of the number of outgoing and incoming links between SNPs for height (blue, top) and diameter (red, bottom) are counted between networks.

Within metagalactic networks, 105 QTLs detected by system mapping resided in only eight different modules, M7 (3 QTLs), M12 (30 QTLs), M66 (6 QTLs), M70 (1 QTLs), M119 (1 QTLs), M151 (15 QTLs), M153 (45 QTLs) and M157 (4 QTLs), implying that the interplay between QTLs located in the same module may govern stem growth. Most QTL-containing modules played as minor roles, with more incoming than outgoing links (Figure 2B). We grouped QTL-containing modules into submodules, then constructed deep intergalactic networks to describe the connections among them. Module M153, which contained 186 SNPs, had the highest number of QTLs of all QTL-containing modules. By comparing BIC values between possible submodule arrangements, the most parsimonious number of submodules was found to be nine. In this intergalactic network organization, SM7/M153 is a minor submodule inhibited by SM2/M153 and SM4/M153 in the height network; however, SM7/M153 regulates other submodules in the diameter network. Although SM4/M153 has a predominant role in the height network, it is regulated by other submodules in the diameter network. From this intergalactic network, QTL-containing submodules exhibited considerable differences in effect on height and diameter, supporting their pleiotropic control of these complex growth traits (Figure 3A).

Local interstellar networks, at the individual SNP level, illustrate specific epistatic distinctions between height and diameter growth. We demonstrated how QTLs and other SNPs that were nonsignificant in the system mapping analysis interact with each other in QTL-containing, local interstellar networks by taking SM5/M153, which is the largest QTL-containing submodule involving 24 QTL in a total of 50 SNPs (Figure 3B), as an example. Based on the independent genetic effects calculated from the CRI differential model, independent local interstellar networks were also constructed (Figure 3C). In all cases, the distribution of outgoing links showed striking differences in numbers, originating from only a small portion of predominant SNPs in these networks. However, all SNPs received incoming links. In general, most QTLs essentially served as receivers, activated or inhibited by other SNPs. We also found that individual QTLs perform differently between the diameter and height networks: for example, in the SM5/M153 height network, QTLs 48806, 48811 and 48892 had the most outgoing links, tending to activate or inhibit other modules; while in the diameter network, the most predominant role was held by SNPs 49614 and 49360, followed by QTLs 48794 and 48806 (Figure 3B). This effect was similar in the independent local interstellar network. In Figure 3C, QTLs 48833 and 48802 were predominant nodes in height growth, whereas QTLs 48891, 48922 and 48886 predominated in diameter growth. In addition, there was a discrepancy in SNP organization between local interstellar networks and independent local interstellar network (Figures 3B,C). On one hand, regulatory roles, especially dominant nodes, change in the network structures. For example, QTL 48806 predominated in the local interstellar height network, while in the independent local interstellar height network, QTL 48833 was predominant. On the other hand, independent networks contained more frequent interactions among SNPs, indicating that the network structure of one trait may be influenced by the growth of the other traits.

**FIGURE 4:**
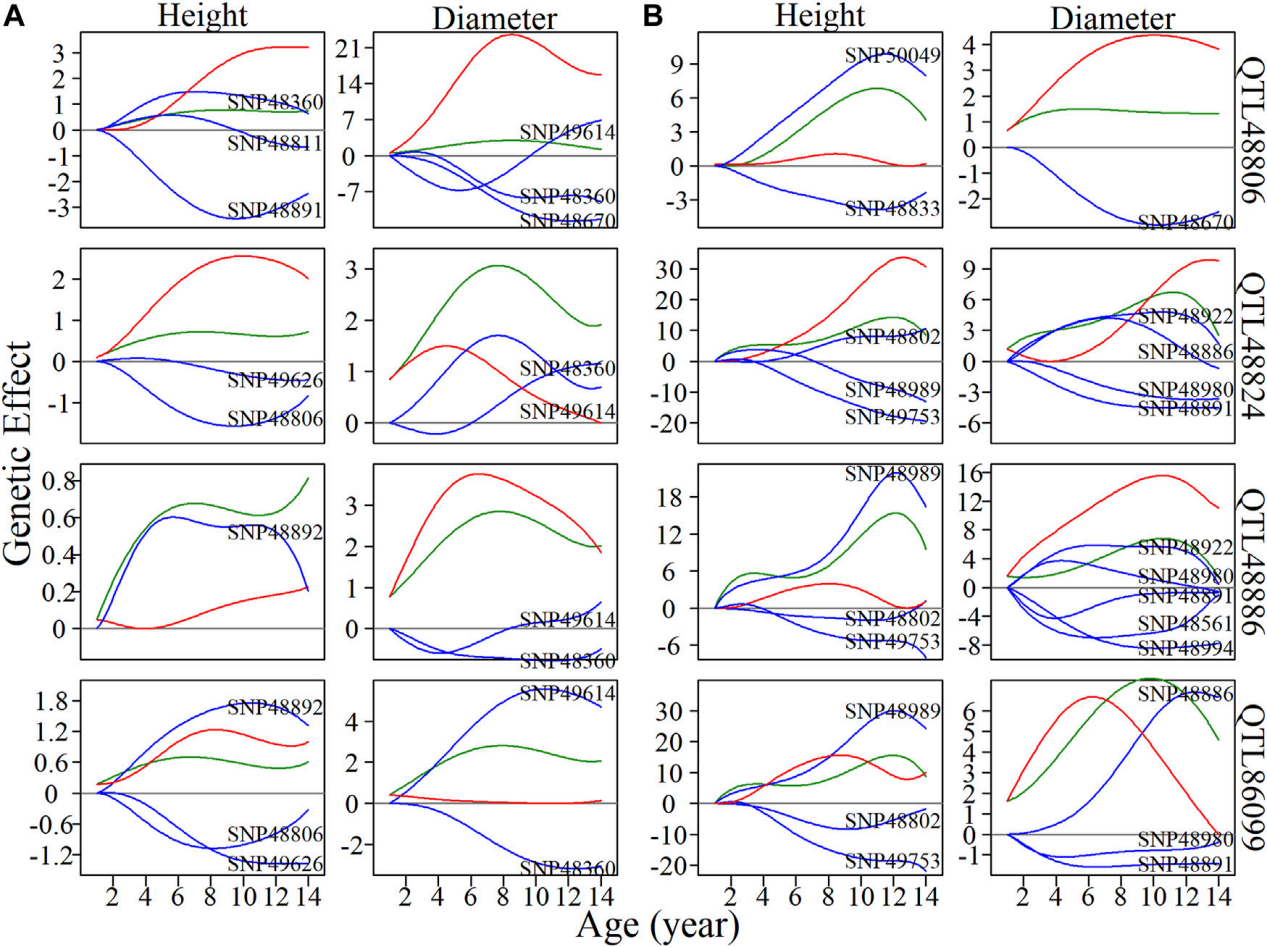
Resolution of quantitative trait locus (QTL) overall and independent effects on stem height and diameter. **(A)** Genetic effect curves and **(B)** independent genetic effect curves of four QTLs from submodule SM5/M153. The net genetic effect of each QTL (green line) is deconstructed into the independent effects (red line) and effects that are dependent on other single nucleotide polymorphisms (SNPs; blue lines).

We selected four QTLs from SM5/M153 to analyze the dynamics of inherent genetic effects and those influenced by other SNPs (Figure 4). QTL 48806 was predominant in both height and diameter networks, and had dramatic, independent effects, and was affected by other SNPs, which exerted an overall negative epistatic effect on this QTL. Thus, the net genetic effects were inferior to the independent effect. In the corresponding independent local interstellar network, a similar pattern of genetic effects was found for diameter, while in the height network, the expression of QTL 48806 is promoted by SNP 50049, and the net genetic effect is larger than the independent effect. We also found that QTL 48886 is upregulated by SNP 48892 in the height network and downregulated by SNP 49614 and SNP 48360 in the diameter network, and the same phenomenon was observed in the independent network. For QTL 86099, the overall genetic effects were similar to the independent effects for height growth, whereas the net genetic effect in diameter growth is greater than expected from its intrinsic capacity. Under independent growth, the diameter net genetic effect is smaller than the independent effect in 1–7 years, but gets stronger after the seventh year.

### Nonsignificant Locus Analysis Within Modules

**FIGURE 5:**
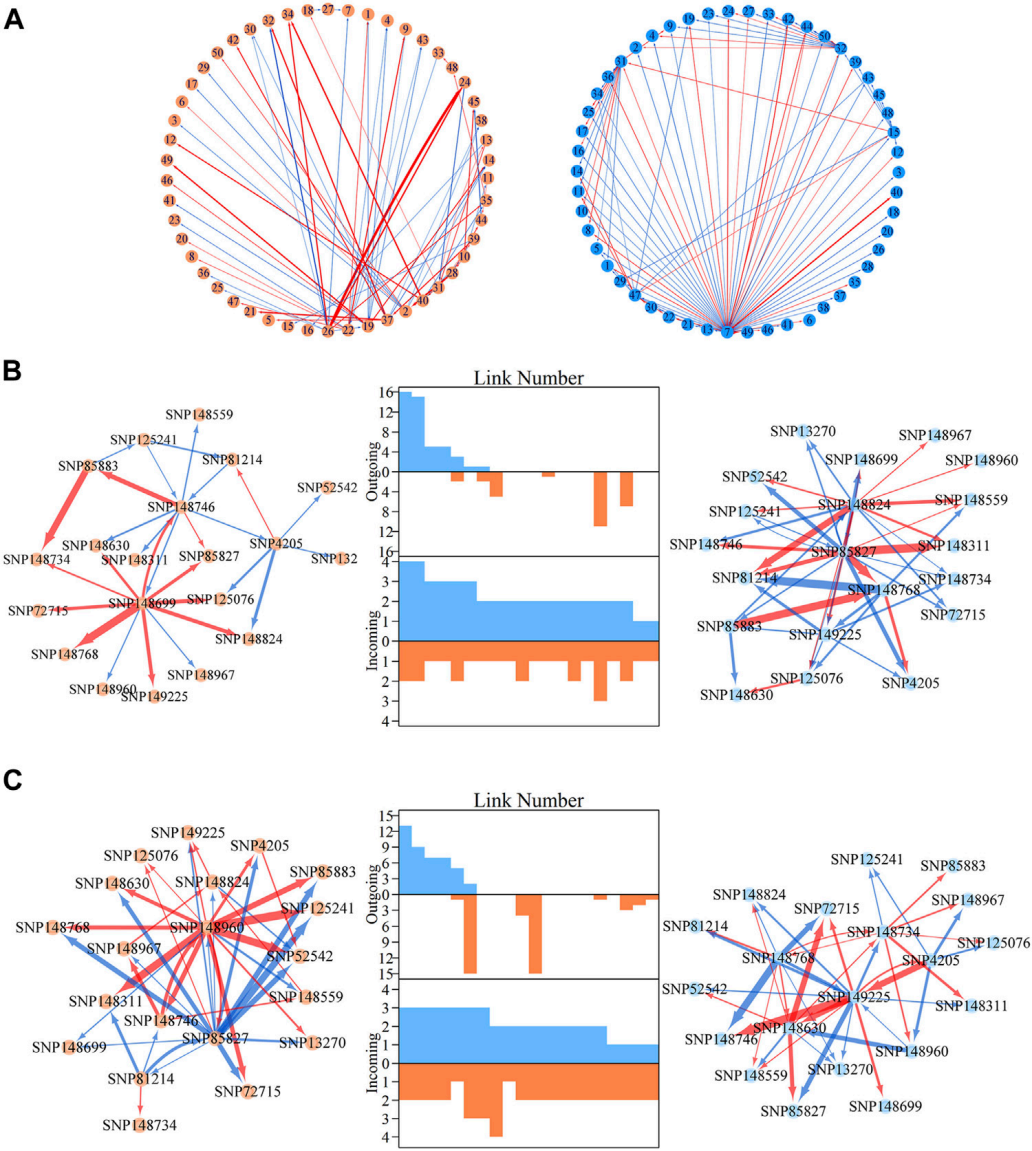
Metagalactic genetic networks of module M85 and intergalactic networks of individual single nucleotide polymorphisms (SNPs) within module SM23/M85 for stem height and diameter. **(A)** Genetic networks among 50 submodules within module M85. **(B)** Genetic networks among 20 SNPs from submodule 23, SM23/M85. **(C)** Independent genetic networks among 20 SNPs from submodule 23, SM23/M85. Red and blue arrowed lines stand for inhibition and activation, with the thickness of lines proportional to the strength of regulation. The distribution of the number of outgoing links and incoming links across SNPs of height (blue, top) and diameter (red, bottom) is counted between networks.

General analysis focused on the pleiotropic control of complex traits by significantly associated SNPs, ignoring those nonsignificant SNPs, which may also have indispensable roles in regulation and control. It is possible that some SNPs have an independent effect, but this effect may be diminished by negative epistatic effects from other SNPs. Here, we randomly chose a module without any significantly associated QTLs, M85, which comprised a total of 565 SNPs. According to the BIC values for clustering, 50 was found to be the most parsimonious number of clusters for the construction of intergalactic genetic networks (Figure 5A). In the height network, directional positive and negative epistasis were found to be basically consistent in numbers of links, while the strength of negative regulation was much greater than for positive regulation, such as in the case of SM24↔SM26. In the diameter network, the directional positive epistatic effect was greater than that of negative epistasis. This indicates submodules tend to compete in their regulation of height growth, but reinforce each other’s effects in regulating diameter growth.

SM23/M85 was found to be a minor submodule in both the height and diameter intergalactic networks, containing 20 SNPs and inhibited by SM22/M85, SM7/M85, and SM32/M85. We constructed overall and independent local interstellar networks for height and diameter (Figures 5B,C). These networks exhibited considerable differences in organization between height and diameter, as revealed by the distribution of outgoing and incoming links in the metagalactic networks. As with the intergalactic networks for M85, directional negative and positive links were approximately equal in the overall height network, and directional positive links outnumbered negative links in the corresponding diameter network. However, this effect disappeared in the independent diameter network, indicating the organizational structure of SNPs differs between independent and interactive perspectives of trait growth.

**FIGURE 6:**
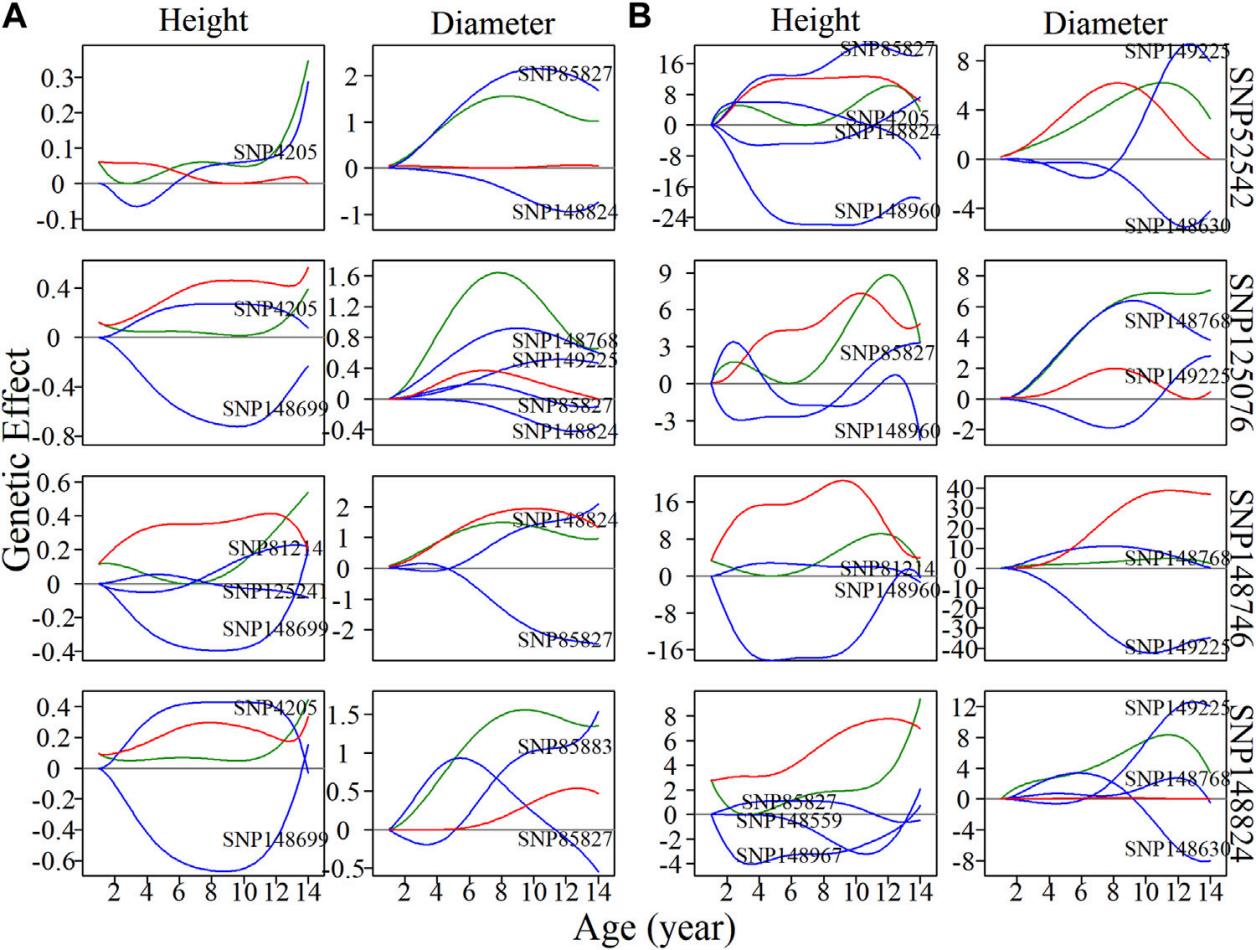
Resolution of single nucleotide polymorphism (SNP) overall and independent effects on stem height and diameter. **(A)** Genetic effect curves and **(B)** independent genetic effect curves of four SNPs from submodule SM23/M85. The net genetic effect of each SNP (green line) is deconstructed into the independent effects (red line) and effects that are dependent on other SNPs (blue lines).

Four nonsignificant SNPs, 52542, 125076, 148746, and 148824 (Figure 6), were found to be located within the local interstellar network of SM23/M85. The overall genetic effect of SNP 52542, located in chromosome 5, on height growth is negatively regulated by SNP 4205 in the first 6 years, and is then upregulated by the same SNP. The net genetic effect on diameter growth was found to be larger than the independent effect. When considering independent effects, SNP 4205 is net-downregulated in height growth, and with net upregulation in diameter growth in the years 11–14. SNPs 125076, 148746 and 148824 were net-downregulated in height growth, in both the overall and independent networks, which offset their considerable independent effects. In contrast, the effects on diameter of SNPs 125076 and 148824 in the overall networks were found to be promoted by other SNPs. Similar effects were also observed for SNP 148824 in the independent diameter growth network: its independent effects were amplified by positive epistasis via incoming links. These examples show that SNPs may exhibit pronounced effects on trait growth if their negative regulators are silenced. However, in some cases, although an SNP may exhibit significant effects on diameter growth, it may also be negatively regulated by other SNPs in height growth, in which case the actual pleiotropic control of complex traits by the SNP is likely to be neglected when traditional analytical approaches are used. The type of regulated SNP, as well as the positivity or negativity and strength of regulation, may vary throughout the growth of different traits, likely acting as an important driver of pleiotropic control.

## Discussion

Research on genetic structures has shown that genes often express pleiotropic effects over two or more traits; this is key to understanding pathways of gene action, assessing potential off-target effects of genetic manipulation with the aim of altering a specific pathway, and comprehending the effects of new mutations on evolution, potentially inducing both favorable and unfavorable effects on fitness (Hill and Zhang, 2012). Genetic mapping and association studies are highly dependent on statistical assumptions integrating reductionist thinking to detect individual, significantly-associated loci (Walling et al., 1998; Kemper et al., 2015; Vanhatalo et al., 2019). However, at the quantitative level, all genes may be pleiotropic in view of the highly interdependent and interactive nature of biological systems; however, until now the interplay pattern of genes is still unclear.

In this paper, we present a computational model integrating a novel growth equation, system mapping, functional clustering, developmental modularity theory, and evolutionary game theory. We established a CRI differential equation to describe the interaction of complex traits (stem height and diameter in forest poplar trees), which can reasonably quantify stem growth and internal structure. By deconstructing the growth of these two traits into a self-regulated part and one that is regulated by interactions between the co-existing traits in the system, our growth model can quantitatively describe the co-operative and antagonistic interactions between these traits in order to generate an in-depth understanding of the whole growth function, and reveal growth potential. We embedded the CRI differential equation into a QTL mapping model to identify important pleiotropic QTLs that play important roles in regulating the growth structure of the traits. On the other side, CRI differential equation provides a useful tool to estimate the net genetic effect of each SNP, which can resolve patterns of change over time based on the mathematical aspect of traits development.

CRI-based simulation studies show that heritability (proportion of genetic variance in the simulated phenotypic variance) and sample size affect mapping precision and power. The simulations are conducted with sample size as 66 (which is equal to the real data), 100 and 200, and heritability as 0.05, 0.1, respectively. For each simulation case, the proportion of simulation times of meaningful QTL screened out from 1,000 genetic markers of repeated simulation experiments is the mapping accuracy (Power). As seen in Supplementary Table S3, the mapping accuracy of QTL detection is above 0.530. The results show that system mapping can reasonably well estimate the time-varying trend of traits growth, even under a modest heritability $\left(H^{2}=0.05\right)$ and a modest sample size (*n* = 66). We also found that the accuracy of effect estimation is sensitive to increasing heritability and sample size. On the other hand, in the absence of QTL expression $\left(H^{2}=0\right)$ , the same genetic sample size of 66, 100, and 200 was simulated with 1,000 genetic marker genes with false positive rates (FPRs) being generally below 0.055 (Supplementary Table S3). According to the QTL mapping accuracy and false positive probability at a series of different thresholds, we expressed ROC curves for different simulated sample sizes and heritability (Supplementary Figure S5). The area under the ROC curve (AUC) was calculated to assess the accuracy of QTL mapping. At the heritability level of 0.1, the AUC of the simulated quantities of the three sample sizes were all relatively high (>0.844). The parameter estimation results and estimated growth curves of different simulation scales are shown in Supplementary Table S4 and Supplementary Figures S6, S7. Our CRI-based QTL mapping has reasonably good statistical properties in interaction detection and FRP controlling.

The most important element of our framework is that the genetic architecture of complex traits is explored from omnigenic, genome-wide perspective (Boyle et al., 2017). One view proposed that the association between genes and traits was represented by a bipartite network and the presence of a modular structure detected by methods developed in physics (Barber, 2007). Our framework model constructed multilayer networks based on functional clustering to discern distinct network modules, in which genes are linked more strongly to each other than to those in other modules. The top layer with the lowest resolution, called the metagalactic network, shows connections between modules; the next layer, the intergalactic network, has increased resolution, shows connection between submodules; and the bottom layer is the local interstellar network, which shows the interaction networks between SNPs and describes directional epistatic interactions. We deconstructed the net effect of genetic loci into independent and dependent effects, describing those in which the effect on complex traits is exerted directly through its own capacity, or indirectly through the regulation of other loci, respectively. The algorithmic aspects of the framework include curve smoothing, variable selection, matrix structuring, and ODE solving, each of which can be improved by introducing advanced theories and modern applied mathematical and statistical methods for future study.

Our model can be applied in general to reconstruct multilayer genetic networks, resolving the effects of genetic interactions and pleiotropy on the development of complex traits. The connection and regulation of the network may change with time or environment (Nie et al., 2017). There is potential extension for allowing a time-varying network instead of static model. In the context of organismal growth, our established framework can be used to further research the interaction of other multidimensional traits. For example, stem growth in trees includes the growth of some lateral organs and branches, in addition to the height and diameter of the stem that we included in our study. The multilayer interactome networks can also be extended from a two-dimensional to a multidimensional trait model, and an interactive regulation network of traits under pleiotropic control could be established, although such expansion will greatly increase the complexity of the model and the difficulty of computing. Our multilayer interactome network provides a robust and reliable modeling framework for assessing gene pleiotropy on traits and the interactions between the development of complex traits.

## Data Availability

The original contributions presented in the study are included in the article/Supplementary Material, further inquiries can be directed to the corresponding authors. The computational code and data that support the findings of this study are available on request from the corresponding author.
