# Supplementary material for: A Multilayer Interactome Network Constructed in a Forest Poplar Population Mediates the Pleiotropic Control of Complex Traits
Source: Front Genet. 2021 Nov 12;12:769688. doi: 10.3389/fgene.2021.769688 (PMC8633413; doi:10.3389/fgene.2021.769688)
Supplement: Supplementary file 1 [file DataSheet2.PDF]

Supplementary Material

1 Supplementary Figures and Tables

1.1 Supplementary Figures

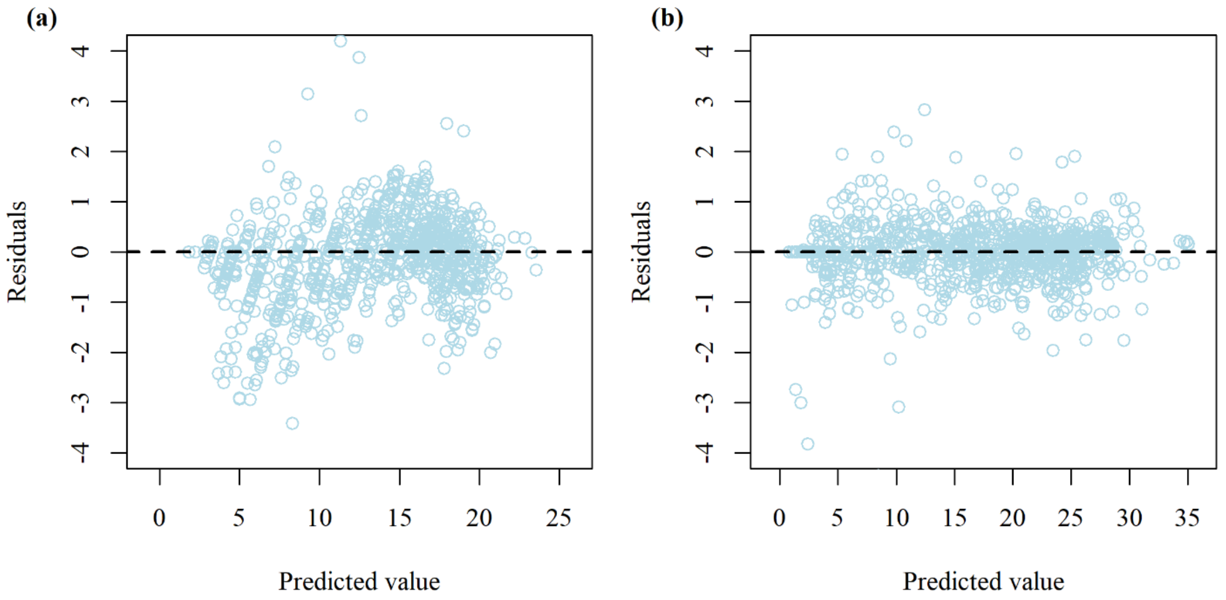

Supplementary Figure 1. Random scatters of residuals over predicted values by the CRI equation of stem height (a) and stem diameter (b) across each tree (circle) of an interspecific full-sib family of Populus, warranting the statistical behavior of data fitting.

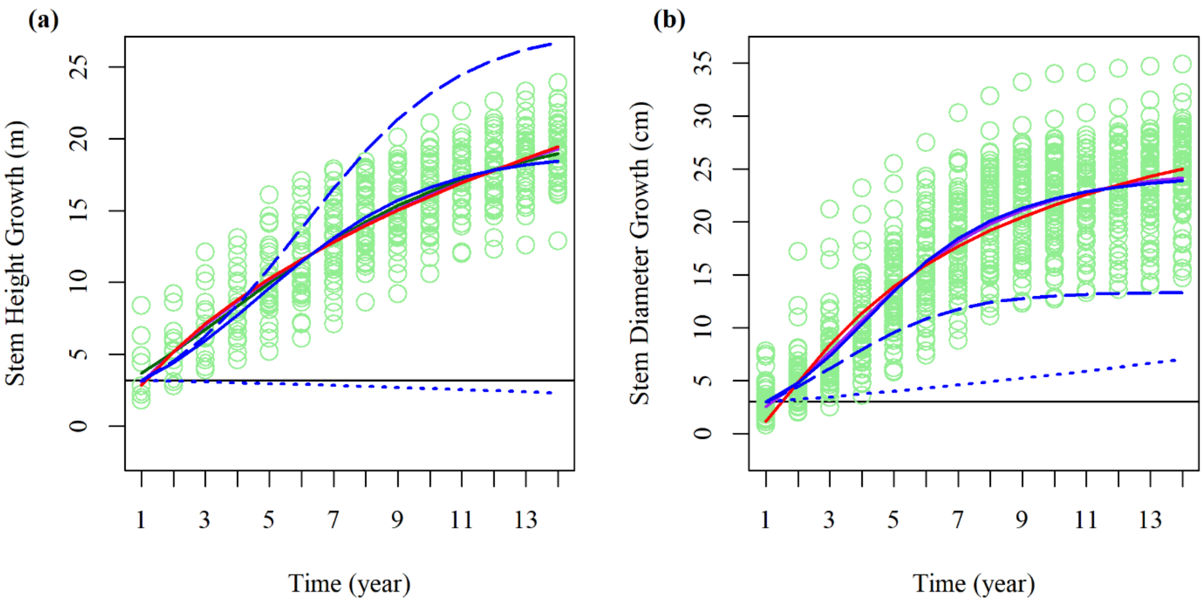

Supplementary Figure 2. The growth trajectories of stem height (a) and diameter (b) of an interspecific full-sib family of Populus in the first 14 years. The green dots represent the phenotypic observations of each sample. Gompertz equation (dark green solid line), Korf equation (red solid line), Richards equation (solid purple line) and CRI equation (solid blue line) fit the mean curves of the two traits. The CRI curves are divided into a potential relatively independent growth part (dashed line) and an interactive growth part regulated by coexisting traits (point line).

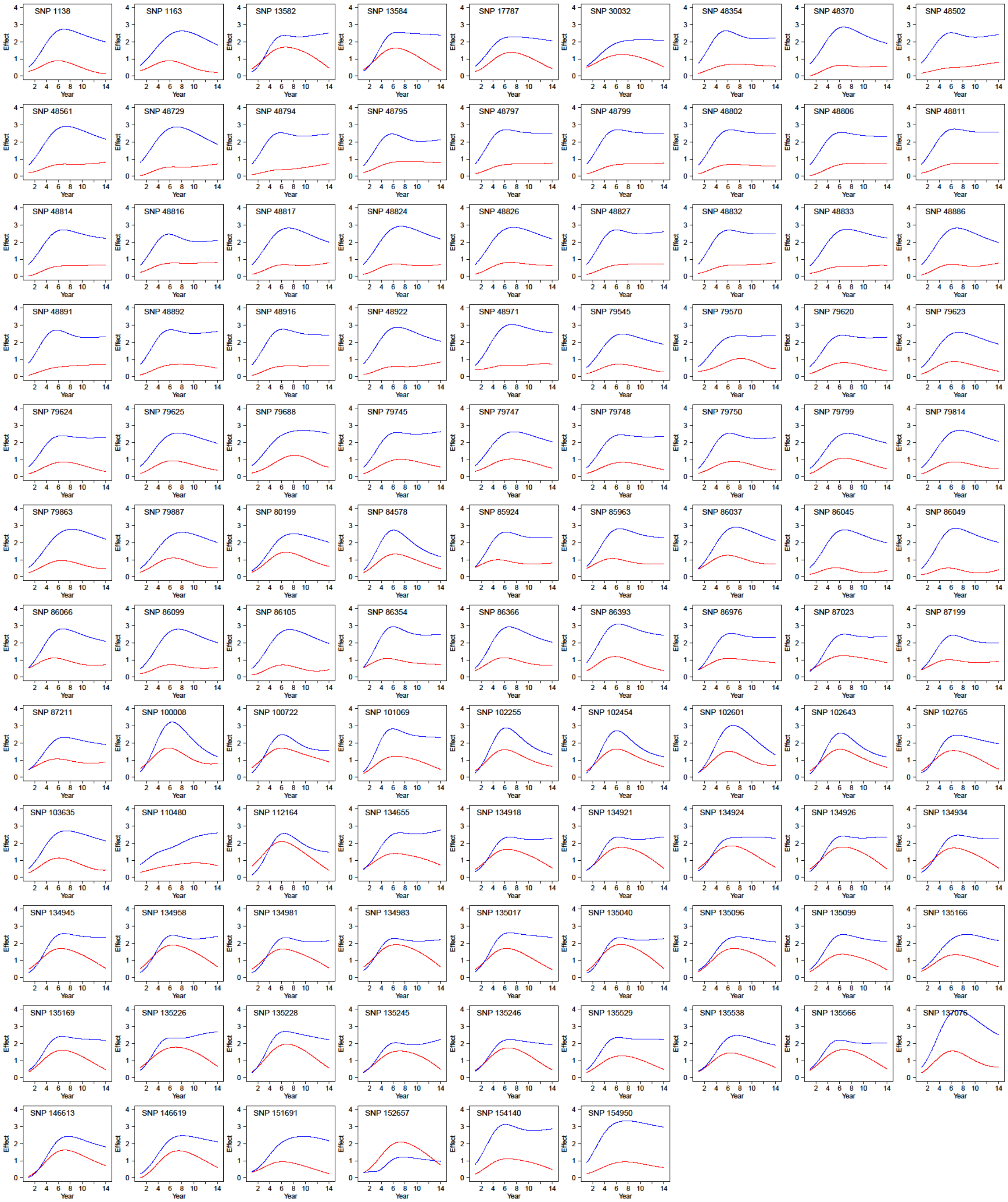

Supplementary Figure 3. Stem height genetic curves (red) and stem diameter genetic curves (blue) of 105 significant SNPs of the Populus genome detected by CRI equation.

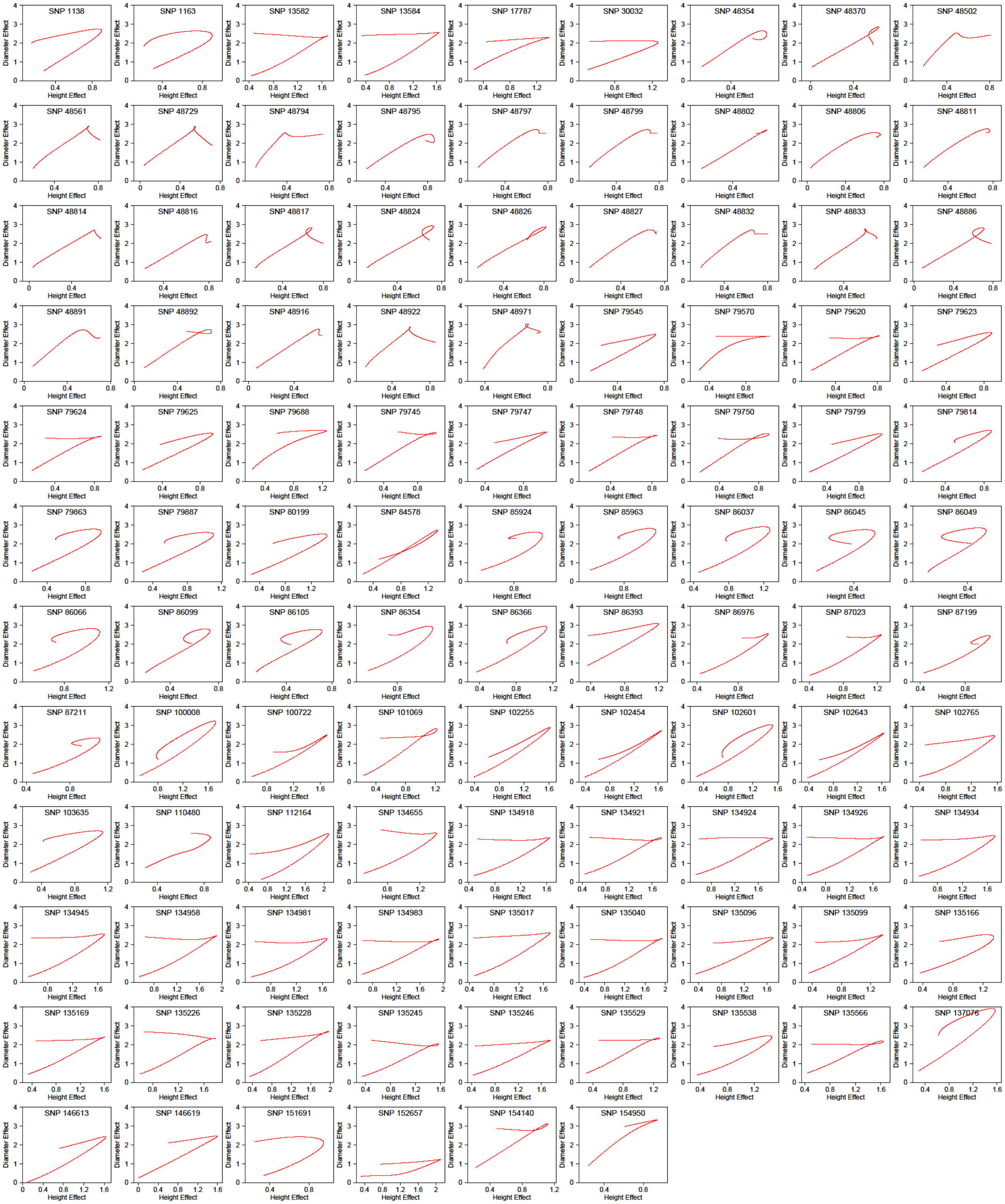

Supplementary Figure 4. The dynamic relationship of genetic effects on stem height and diameter growth of 105 significant SNPs of the Populus genome detected by CRI equation.

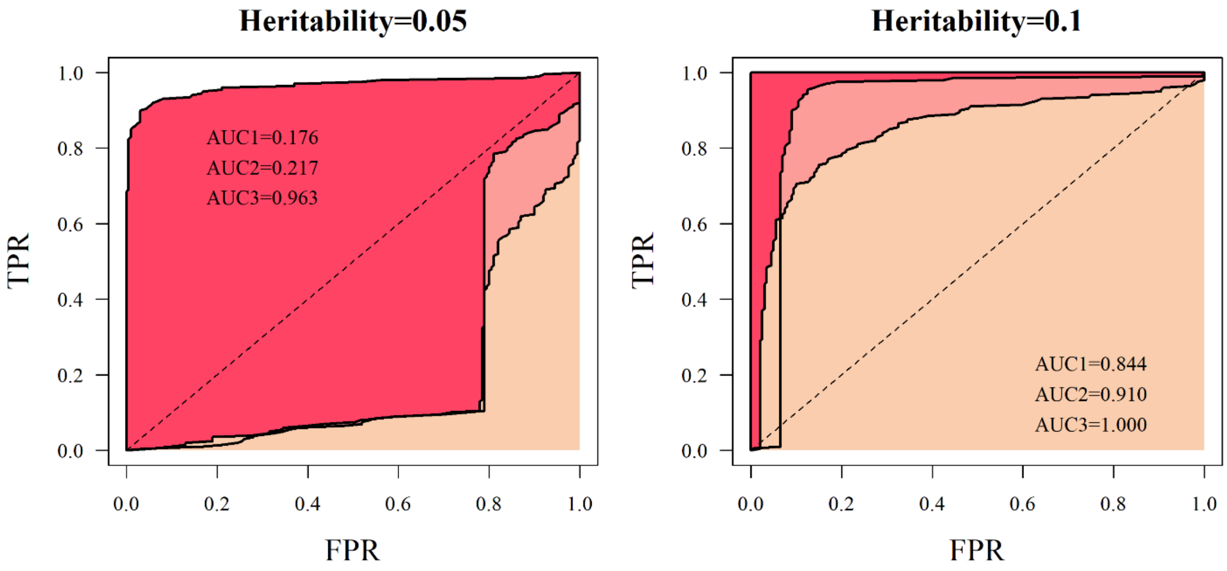

Supplementary Figure 5. ROC curves from simulation under sample size 66, 100, 200 and heritabilities 0.05 and 0.1 respectively, and the calculated AUC values are shown on the graphs. AUC1 represents the area under the ROC curve with a sample size of 66, AUC2 represents the area under the ROC curve with a sample size of 100, and AUC3 represents the area under the ROC curve with a sample size of 200.

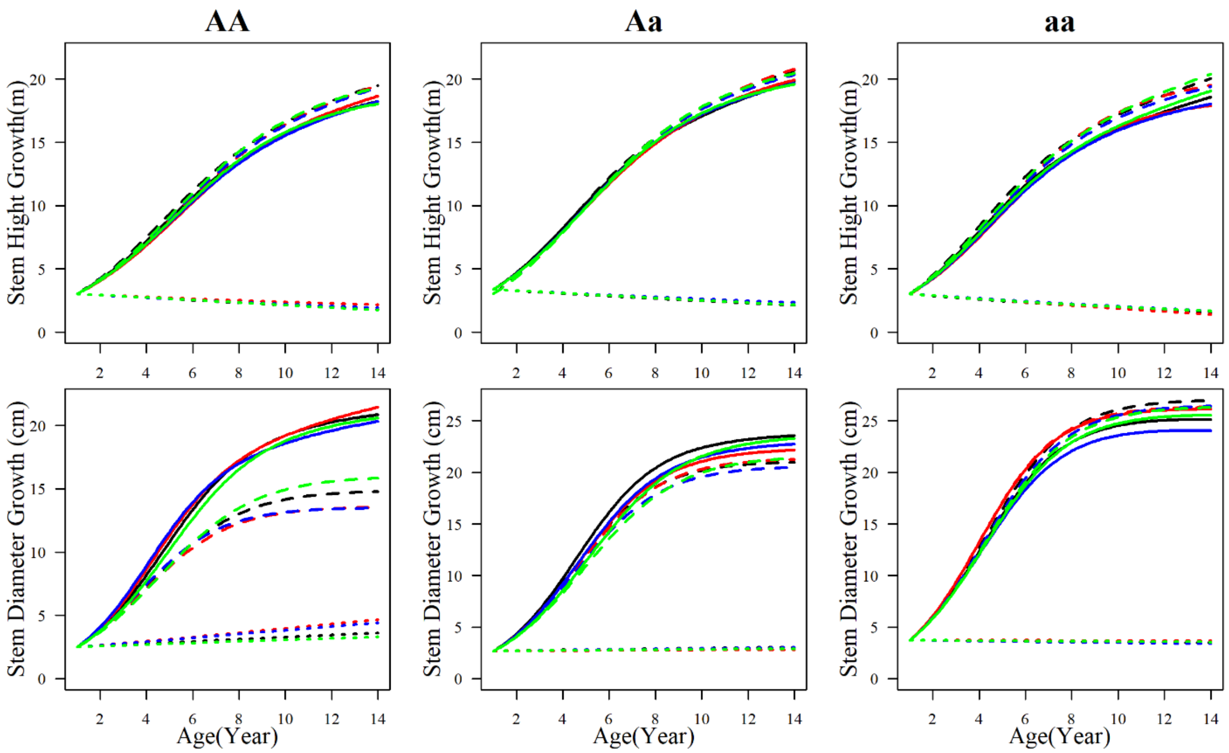

Supplementary Figure 6. Estimated curves of genotype AA, Aa and aa from simulation under heritability 0.05 and the sample size 66,100,200, in comparison with true curves. Black represents the true curve, red represents the estimation curve under the sample size of 66, blue represents the estimation curve under the sample size of 100, and green represents the estimation curve under the sample size of 200. The overall growth (solid line) for each trait is decomposed into its independent (broke line) and interactive growth components (dot line).

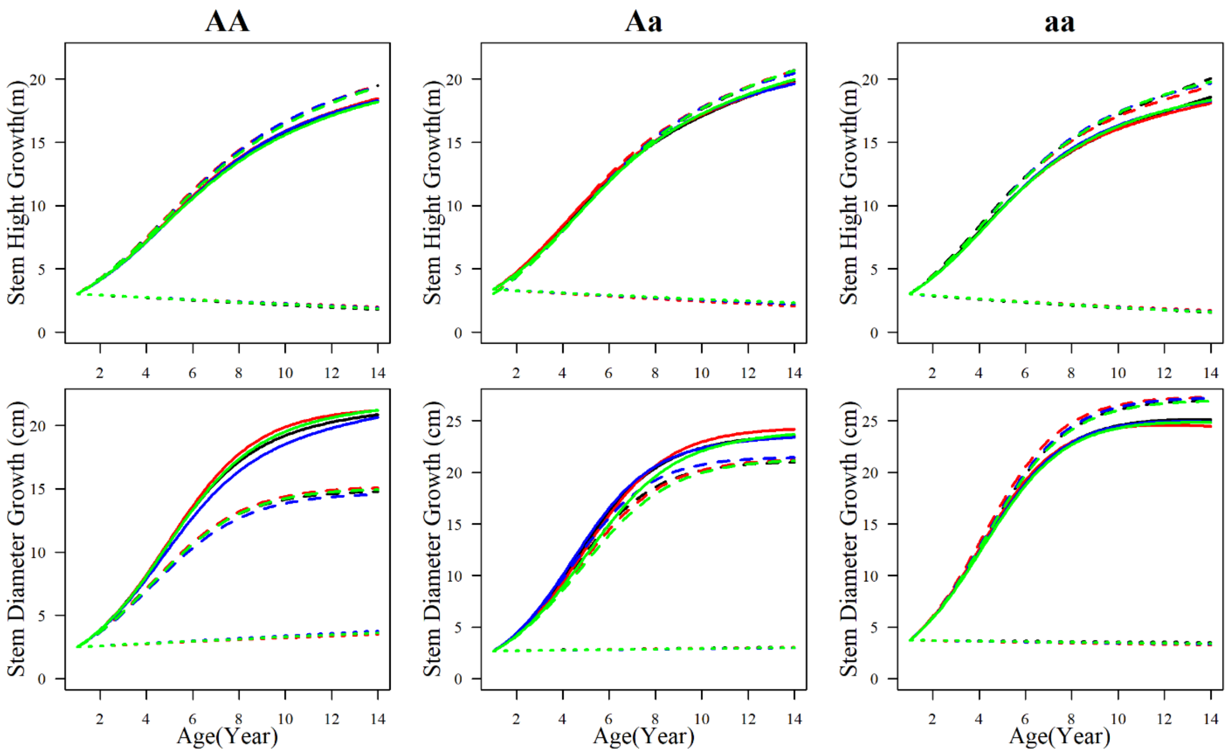

Supplementary Figure 7. Estimated curves of genotype AA, Aa and aa from simulation under heritability 0.1 and the sample size 66,100,200, in comparison with true curves. Black represents the true curve, red represents the estimation curve under the sample size of 66, blue represents the estimation curve under the sample size of 100, and green represents the estimation curve under the sample size of 200. The overall growth (solid line) for each trait is decomposed into its independent (broke line) and interactive growth components (dot line).

1.2 Supplementary Tables

Supplementary Table 1. Estimated parameters of four equations of Gompertz (G), Korf (K), Richards (R) and CRI equation fitting for stem height and diameter of full-sib family of Populus, and the evaluation information: Akaike information criterion (AIC), Schwarz criterion (SC), hannan-quinn criterion (HQ).

G		K		R		CRI	
Height	Diameter	Height	Diameter	Height	Diameter	Height	Diameter
K = 21.23	K = 24.89	K =414.15	K =42.47	K =28.44	K=25.15	α_H=0.41	α_D=0.54
a= 0.77	a=1.15	b=4.97	b=3.59	a=0.99	a=0.43	K_H=27.75	K_D=13.35
b= 0.21	b=0.33	c=0.18	c=0.72	b=0.07	b=0.30	$\beta_{H\leftarrow D}$=-0.01	$\beta_{D\leftarrow H}$=0.04
				m=0.88	m=6.18		
AIC=11.85		AIC=10.37		AIC=10.12		AIC=5.55	
SC=12.05		SC=10.57		SC=10.38		SC=5.75	
HQ=11.80		HQ=10.32		HQ=10.05		HQ=5.50	

Supplementary Table 2. Detailed information and annotations of significant SNPs detected by CRI equation (separate Excel file).

Supplementary Table 3. Mapping accuracy and false positive probability of CRI-based QTL mapping were evaluated by computer simulation under different simulation scales with heritability of 0,0.05, 0.1 and sample size of 66,100,200, respectively.

Heritability	Size	66	100	200
FPR	0	0.045	0.055	0.040
Power	0.05	0.530	0.740	0.920
	0.1	0.735	0.970	1.000

Supplementary Table 4. Estimated CRI equation parameters obtained from 100 simulations at a set locus with AA, Aa, and aa genotypes under the heritability levels of 0.05 and 0.1 and the sample size as 66, 100 and 200.

parameter			α_H	K_H	$\beta_{H\leftarrow D}$	α_D	K_D	$\beta_{D\leftarrow H}$
True		AA	0.3757	96.2408	-0.0358	0.5078	14.8799	0.0232
		Aa	0.3849	119.3091	-0.0323	0.5631	21.0653	0.0065
		aa	0.4442	132.5970	-0.0315	0.5688	27.0662	-0.0038
$H^2 = 0.05$	Size=66	AA	0.3108 (0.1019)	80.2977 (2.3048)	-0.0309 (0.0068)	0.4933 (0.2451)	12.7444 (4.7949)	0.0606 (0.0723)
		Aa	0.3456 (0.0434)	82.0137 (3.0576)	-0.0294 (0.0022)	0.4344 (0.0606)	15.9334 (5.2229)	0.0395 (0.0372)
		aa	0.3585 (0.0852)	83.7953 (2.8082)	-0.0264 (0.0030)	0.4972 (0.0800)	37.9505 (15.3849)	-0.0159 (0.0111)
	Size=100	AA	0.3358 （0.0648）	80.9130 （0.9973）	-0.0336 （0.0025）	0.4229 （0.0991）	16.6422 （4.4153）	0.0276 （0.0460）
		Aa	0.3526 （0.0452）	81.8497 (2.5755)	-0.0296 (0.0019)	0.4323 (0.0547)	18.2358 (3.3859)	0.0200 (0.0172)
		aa	0.3427 (0.0696)	82.3898 (1.7492)	-0.0266 (0.0029)	0.4866 (0.0594)	30.6838 (4.8501)	-0.0091 (0.0068)
	Size=200	AA	0.3273 (0.0512)	82.0519 (1.6787)	-0.0329 (0.0031)	0.4802 (0.1907)	15.1305 (5.7578)	0.0454 (0.0665)
		Aa	0.3414 (0.0299)	83.7266 (5.7865)	-0.0290 (0.0014)	0.4252 (0.0370)	20.1983 (2.8525)	0.0124 (0.0097)
		aa	0.3438 (0.0583)	83.6358 (2.7349)	-0.0273 (0.0017)	0.4907 (0.0338)	30.4847 (5.0473)	-0.0087 (0.0065)
$H^2 = 0.1$	Size=66	AA	0.3546 (0.0635)	81.4860 (1.9673)	-0.0339 (0.0030)	0.4320 (0.0976)	14.3883 (5.3133)	0.0473 (0.0635)
		Aa	0.3753 (0.0339)	83.1607 (4.1430)	-0.0296 (0.0013)	0.4571 (0.0499)	19.8523 (3.3771)	0.0131 (0.0129)

	aa	0.3776<br>(0.0417)	82.3333<br>(2.2279)	-0.0276<br>(0.0019)	0.5445<br>(0.0632)	30.8809<br>(5.8694)	-0.0098<br>(0.0078)
Size=100	AA	0.3500<br>(0.0469)	82.0428<br>(3.3255)	-0.0328<br>(0.0019)	0.4193<br>(0.0578)	16.7964<br>(3.5566)	0.0207<br>(0.0226)
	Aa	0.3602<br>(0.0259)	82.2530<br>(2.0574)	-0.0292<br>(0.0012)	0.4561<br>(0.0496)	18.4031<br>(4.5085)	0.0238<br>(0.0309)
	aa	0.3839<br>(0.0594)	82.7795<br>(2.1869)	-0.0277<br>(0.0015)	0.5320<br>(0.0388)	31.1368<br>(4.5686)	- 0.0109<br>(0.0068)
Size=200	AA	0.3536<br>(0.0295)	82.6057<br>(1.6231)	-0.0339<br>(0.0014)	0.4324<br>(0.0540)	16.4372<br>(3.4408)	0.0200<br>(0.0184)
	Aa	0.3620<br>(0.0146)	85.2817<br>(8.9775)	-0.0293<br>(0.0009)	0.4645<br>(0.0226)	21.0377<br>(2.3310)	0.0082<br>(0.0075)
	aa	0.3671<br>(0.0350)	83.2900<br>(2.2469)	-0.0275<br>(0.0010)	0.5382<br>(0.0325)	29.9876<br>(5.7658)	-0.0077<br>(0.0087)
